# Valorization of Hemp Core Residues: Impact of NaOH Treatment on the Flexural Strength of PP Composites and Intrinsic Flexural Strength of Hemp Core Fibers

**DOI:** 10.3390/biom10060823

**Published:** 2020-05-27

**Authors:** Fabiola Vilaseca, Ferran Serra-Parareda, Eduardo Espinosa, Alejandro Rodríguez, Pere Mutjé, Marc Delgado-Aguilar

**Affiliations:** 1 Advanced Biomaterials and Nanotechnology, Department of Chemical Engineering, University of Girona, 17003 Girona, Spain; fabiola.vilaseca@udg.edu; 2 Department of Industrial and Materials Science, Chalmers University of Technology, SE 412 96 Gothenburg, Sweden; 3 LEPAMAP Research Group, University of Girona, Maria Aurèlia Capmany, 61, 17003 Girona, Spain; ferran.serrap@udg.edu (F.S.-P.); pere.mutje@udg.edu (P.M.); m.delgado@udg.edu (M.D.-A.); 4 Chemical Engineering Department, Bioagres Group, Faculty of Science, Universidad de Córdoba, Building Marie-Curie, Campus of Rabanales, 14071 Cordoba, Spain; eduardo.espinosa@uco.es; 5 Chair on Sustainable Industrial Processes, University of Girona, Maria Aurèlia Capmany, 61, 17003 Girona, Spain

**Keywords:** hemp core, chemical treatment, composites, flexural strength, intrinsic properties

## Abstract

Hemp core is a lignocellulosic residue in the production chain of hemp strands. Huge amounts of hemp core are gathered annually in Europe (43,000 tons) with no major application end. Such lignocellulosic wastes have potential as filling or reinforcing material to replace synthetic fibers and wood fibers in polymer composites. In this study, hemp core biomass was treated under different NaOH concentrations and then defibrated by means of Sprout Waldron equipment to obtain single fibers. Polypropylene matrix was reinforced up to 50 wt.% and the resulting hemp core fibers and the flexural properties were investigated. The results show that the flexural strength of composites increased with the intensity of NaOH treatment. The effect of NaOH was attributed to the removal of extractives and lignin in the fiber cell wall leading to improved interfacial adhesion characteristics. Besides, a methodology was established for the estimation of the intrinsic flexural strength of hemp core fibers. The intrinsic flexural strength of hemp core fibers was calculated to be 940 MPa for fibers treated at 10 wt.% of NaOH. In addition, a relationship between the lignin content and the intrinsic strength of the fibers was established.

## 1. Introduction

During the last decade, the incorporation of natural fibers as reinforcing elements in polymer composites has received considerable attention in the academic and industrial field. Interest is warranted due to the advantages of natural fibers over current fossil-based reinforcement such as glass fibers, including low environmental impact, low specific weight, low cost, less damage to manufacturing equipment, minimal health hazards, and reasonably high mechanical properties [1,2,3,4,5,6]. From an economic and environmental perspective, lignocellulosic residues coming from plant fibers manufacturing provides a great opportunity to avoid the use of other agricultural lands and woody resources. In fact, the use of lignocellulosic wastes from the industrial processing of plants as reinforcement in composite materials is one of the most important targets in recent material research [7,8,9,10,11,12].

**Figure 1:**
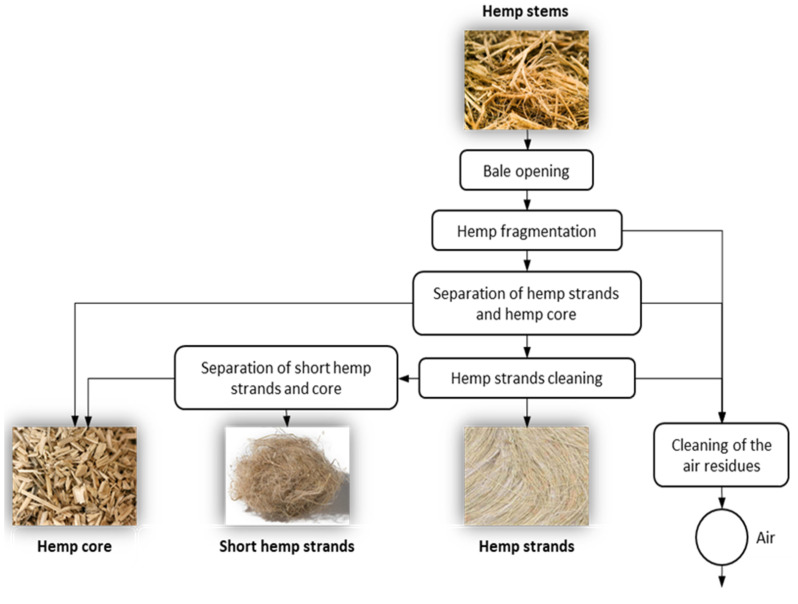
Industrial processing of hemp stems.

Industrial hemp core (HC) is a lignocellulosic residue with no major end application in the production chain of hemp strands [13]. In contrast to the high quality of hemp strands, hemp core is the least valuable part of the plant, but with similar chemical composition to wood [14]. Roughly, hemp stems are constituted by 30–35% of strands, 50–55% of hemp core, and 10–15% of dust/impurities. As a result, the industrial processing aiming at the separation of hemp strands from the stem of the plant contributes to generate huge amounts of hemp core. According to the European Industrial Hemp Association (EIHA), in Europe, where 85% of the world hemp cultivation is located, about 85,000 tons of hemp are harvested and processed yearly. From these, 25,000 tons of strands are delivered as the added-value product. Hemp strands have some of the best mechanical properties among natural fibers, making them attractive in various sectors such as pulp and paper industry, textile industry, biocomposites production (focused in the automotive sector), and insulation materials [15]. Besides, 43,000 tons constitute the woody core of the stem and are considered as residues of the process. The dust and impurities are also residues of the processing chain, a major part of which is pelletized for incineration, while the rest goes for compost and other uses. Figure 1 shows the production chain of hemp strands with the subsequent isolation of hemp core.

After harvesting, hemp plants are treated to extract the strands from the stem of the plant. Initially, the plant is fragmentated obtaining a mixture of strands and core. Then, the strands are separated from the fibrous mixture and later cleaned, whereas the hemp core remains as residue of the production chain.

During the separation of the strands from the stem, the elevate content of hemp core in the hemp stem has a major impact on the manufacturing costs. Such costs can vary depending upon the content of core in the initial biomass. From an economic and social viewpoint, the further use of this residue in other applications would add value to hemp core and to the overall industrial processing of hemp. This could finally make the harvesting of this plant an important factor influencing the industrial and social development of the geographic places where it is cultivated. Environmentally, using a residue coming from an industrial process with the final purpose of adding value is the goal of the circular economy [16,17] and is also in line with the principles of green chemistry [18].

Currently, hemp core residues are aimed to high performance bedding materials for animals, with a market share of 63%. Another interesting new and increasing market for hemp core is in the construction sector, usually combined with lime, where the market share is 16%. Less representative sectors for hemp core fibers are garden much, fungi cultivation and incineration [19].

In recent years, a growing interest in using hemp core as additive in polymer composites has been recorded. Interesting properties such as thermal, mechanical, acoustic, low density, low cost, and sustainability are determining in its function in composites. Regarding mechanical properties, hemp core fibers have been usually considered as filler rather than reinforcement, acting more as a stiffening agent. This is explained by the weak interfacial adhesion between the polymer and the fiber, which lead to poor capacity of transferring the stress throughout the material [20,21].

The two main drawbacks affecting the quality at the interphase of hemp core fibers in composites are its chemical composition, owing to the elevate contents of lignin (21–24%), and low aspect ratios (length/diameter). Although there is an increasing concern on the improvement of the interfacial bonding strength in the global field of natural fiber composites via either fibers modification or by using compatibilizers [22], the potential effect of chemically treated hemp core fibers on the enhancement of the interfacial adhesion has not been widely analyzed.

If the purpose is to open a new market for hemp core fibers in polymer composites, the issue of the interfacial adhesion must be solved. To this end, alkaline treatments such as sodium hydroxide (NaOH) pulping can help the removal of lignin and increase the cellulose content. Additionally, if the digested fibers are passed through a mechanical defibrator, it is possible to achieve a better individualization of the fiber, and therefore higher aspect ratios. Lignin derived from fibers digestion process is garnering interest in the field of composite materials as a potential source of carbon fiber, with the final goal of adding value to the lignocellulosic residue [23,24,25]. Lignin is also considered a potential bioresource in other fields such as concrete additives, thermoplastic materials, and fuel production, among others [26,27].

Indeed, the different chemistry of polymer and natural fibers generally conduct to poor compatibility between the phases. Thereby, the present work proposes the addition of a coupling agent to enhance the interfacial adhesion. More specifically, malleated polymers promote the adhesion by forming linkages with fibers hydroxyl groups and with the unmodified polymer chains [28,29,30].

When a new material is developed and introduced in the market, it is of great importance that it meets the industrial demands required for a certain application. Subsequently, the potential of composites materials is mainly determined by their mechanical properties. Specifically, flexural properties are of great interest and relevance for engineers when envisioning the potential of the material for structural, semi-structural, construction, and other similar market sectors. This is explained by the fact that flexural/bending conditions are very common, whereas purely tensile cases are scarce in comparison. This makes designers especially interested in predicting the behavior of such materials under flexural loads [31]. By the analysis of the flexural properties of the composites, it is possible to model the behavior of composites for different reinforcing amounts, allowing the acquisition of the fiber’s intrinsic flexural strength and compatibility degree as main important outcomes. To the best of our knowledge, the macro and micro flexural properties of hemp core composites have not yet been investigated.

Overall, the current investigation aimed at preparing composites at different reinforcement amounts based on chemically treated hemp core fiber and polypropylene (PP). PP was selected in this study as the polymer matrix, since polyolefins still suppose the largest percentage of polymer used in the composite field, where PP is the most representative. Additionally, the chemical resistance, great mechanical properties, low density, and excellent processability of PP confers the polymer a wide range of applicability [21]. Malleated polypropylene (MAPP) was added in the formulation to enhance the interfacial strength and solve the incompatibilities between the phases. Mechanical properties were evaluated with special attention to behavior under flexural conditions. Finally, the flexural properties were modeled by means of micromechanical models to deduce the intrinsic flexural strength of hemp core fibers.

## 2. Materials and Methods

### 2.1. Materials

Composite materials were prepared using polypropylene (PP) as the polymer matrix and hemp core fibers as reinforcement. The untreated hemp core fibers were provided by Agrofibra S.L. (Puigreig, Spain). Polypropylene PP ISPLEN 090 GDM was supplied by Repsol S.A. (Tarragona, Spain), with a melt flow index of 30 g/10 min (at 230 °C, 2.16 kg) and density of 0.905 g/cm^3^ in accordance with the manufacturer’s data. Maleic anhydride polypropylene (MAPP) was added to the formulation as coupling agent. MAPP was Epolene G 3015 (Eastman Chemical, Middelburg, The Netherlands) with a density of 0.913 g/cm^3^.

All reagents used in this investigation were provided by Sigma-Aldrich and used as received.

### 2.2. Methods

#### 2.2.1. Hemp Core Fibers Treatment

The hemp cores were introduced in a pressurized reactor for 90 min at 98 ± 2 °C. The hemp cores were treated at three different concentrations of caustic soda (NaOH) to evaluate how the extent of the treatment affected the fibers performance. For this, hemp core was submitted to three different treatments at 5%, 7.5%, and 10% of NaOH. Additionally, anthraquinone was added to each batch at a 0.1% with respect to fiber content. Anthraquinone acts as catalyzer during the chemical degradation of lignin. The digestions were carried out at solid to liquid ratios of 1:10. The temperature, duration, and solid to liquid ratio were selected owing to the authors’ expertise in the field of natural fibers and processing techniques. Besides, the different concentrations of NaOH were selected for the following reasons: (i) minimization of residues during the processing of hemp core with the aim of contributing to sustainable methodologies; amd (ii) improvement of the composites’ properties and fibers’ intrinsic flexural strength. Harsher treatments over 10 wt.% of NaOH may cause excessive delignification and damage the fiber cell wall, resulting in a reduction of intrinsic properties.

The digested fibers were then washed profusely with deionized water. Afterwards, the pulp was passed though mechanical defibration Sprout Waldron model 105-A (Muncy, PA, USA) to obtain single fibers. Finally, the fibers were oven-dried at 80 °C until constant weight.

The kappa number of the obtained fibers was measured following TAPPI T 236 om-06. Morphological characteristics of the fibers such as the length (l^f^) and diameter (d^f^) were determined with a MorFi compact device analyzer (Techpap, Grenoble, France). The equipment allows the acquisition of 30,000 images in each test. Four samples of each type of fiber were analyzed.

#### 2.2.2. Composites Preparation and Sample Obtaining

Polypropylene and hemp core fibers were blended at weight ratios of 90/10, 80/20, 70/30, 60/40, and 50/50 (polymer/reinforcement). The blends were processed by means of a Gelimat kinetic mixer model G5S (Draiswerke, Mahaw, NJ, USA). The fibers were initially added to the mixer at 300 rpm speed, and then the polymer and the coupling agent were added by maintaining speed constant. Thereafter, the speed was increased to 2500 rpm and kept until complete melt of the polymer (approximately after 2 min). The mixture was then discharged and cooled down at room temperature. The composite was palletized by means of a blade mill equipped with a 5-mm mesh. Finally, the material was kept in the oven at 80 °C until further processing.

Normalized specimens for the mechanical test were produced with a steel mold in an injection molding machine Aurburg 220 M 350–90U (Aurburg, Loßburg, Germany). For the determination of the intrinsic flexural strength of the fibers, the tensile properties are required. Consequently, flexural and tensile specimens were produced.

#### 2.2.3. Mechanical Test

Prior to testing, the specimens were kept in a conditioning chamber (Dycometal, Sant Boi de Llobregat, Spain) at 23 °C and 50% relative humidity for 48 h, in agreement with ASTM D618 standard. Mechanical tests were conducted by means of an Instron universal testing machine fitted with a 5 kN load cell (Instron, Cerdanyola del Vallès, Spain). Flexural and tensile properties were measured according to ASTM D790 and ASTM D638, respectively. At least five specimens of each composite formulation were tested.

Once the flexural and tensile properties of the composites were evaluated, a micromechanical analysis was performed. The used models are further discussed in the results section.

**Figure 2:**
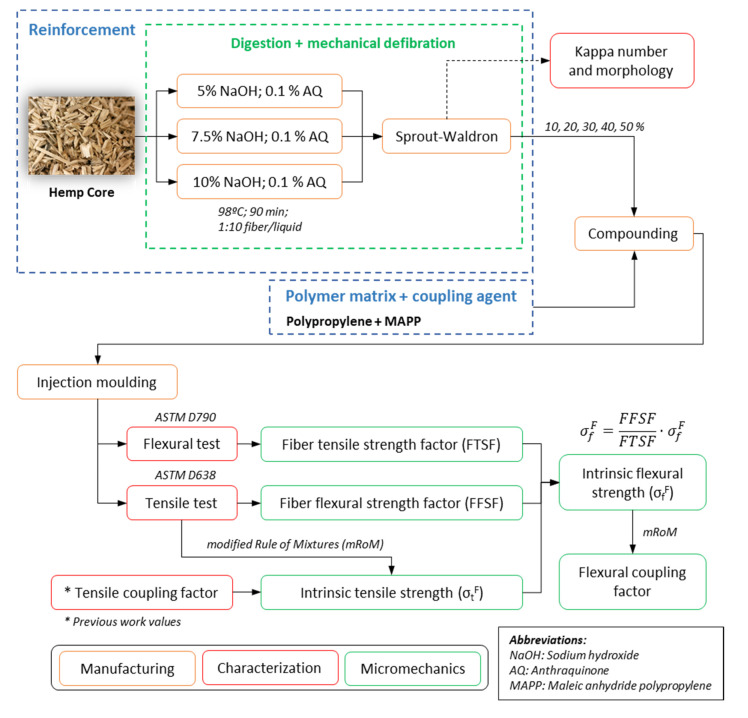
Flowchart of the present investigation.

Towards a better understanding on the methodology and characterization at a macro and micro scale, Figure 2 schematically shows the workplan of the current investigation.

## 3. Results

### 3.1. Effect of Sodium Hydroxide (NaOH) Treatment on Hemp Core Fibers

The fiber cell wall is mainly constituted by cellulose, hemicelluloses, and lignin. The outer layers of the cell wall, between the middle lamella and the primary wall, contain high percentages (≈70%) of lignin. Here, lignin acts as cementing material between fibers [32]. Soft sodium hydroxide (NaOH) treatments aims at the removal of lignin to increment the exposure of cellulose and hemicellulose in the fiber surface.

Furthermore, a defibration step was added after fibers’ digestion. In this context, the degradation of lignin may help the individualization of single fibers getting higher aspect ratios (length/diameter). It is worth mentioning that untreated virgin hemp core biomass was impossible to individualize, attaining only large amounts of fiber bundles and low aspect ratios.

The lignin content and morphology of hemp core fibers was evaluated by measuring the kappa number, the mean fiber length, and fiber diameter. The processing yield was also computed as indicator of the mass loss throughout the process (Table 1).

With the NaOH concentration, the kappa number decreased, indicating lignin loss during the treatment. The kappa number of the untreated hemp core biomass accounted for 95.3 ± 0.6. It is concluded that the treatment offered an effective lignin removal. Indeed, the kappa number decreased linearly with the NaOH concentration with a correlation factor of R^2^ = 0.987. The greater is the loss of matter, the lower is the processing yield; however, the processing yields were still high enough to be considered as high-yield treatments. Such yields ensure the minimum generation of residues during the processing of the lignocellulosic material, agreeing with the principles of green chemistry [18]. Consequently, submitting biomass to higher NaOH concentrations was discarded as the generation of residues would be unreasonable. Moreover, higher NaOH percentages can cause excess delignification damaging or weakening the fiber cell wall, contributing to a decrease of fiber length and aspect ratio [33].

Besides, the aspect ratio increased with the intensity of the NaOH treatment. As previously stated, the degradation and removal of lignin at the outer layers help the individualization of the fibers, achieving larger aspect ratios. The increase of the aspect ratios was mainly attributed to the rise of the fiber length, since the diameter remained almost unchanged.

### 3.2. Optimization of the Coupling Agent

In general, the flexural strength of composite materials depends on the type and amount of reinforcement, the dispersion grade, distribution into the matrix, the aspect ratio of the fibers (*l/d*), and largely on the adhesion at the fiber–matrix interface [28,34]. Nonetheless, lignocellulosic materials are hydrophilic in nature due to an abundance of hydroxyl groups. This makes the fiber incompatible with hydrophobic matrices. This incompatibility leads to poor capacity of transferring the stress through the reinforcement, hindering the increment of the flexural strength by the addition of any lignocellulosic element.

**Figure 3:**
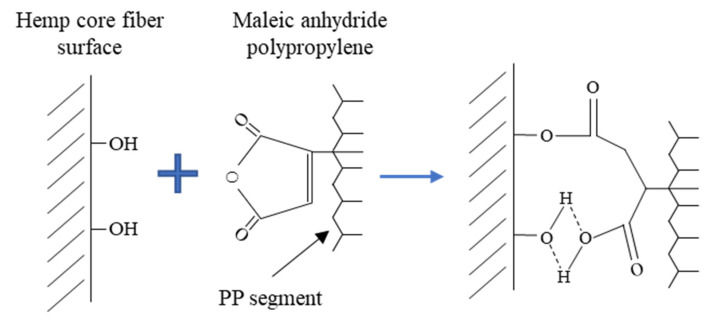
Illustration of MAPP’s interaction with hydroxyl groups at fiber’s surface.

One feasible and effective method to improve the interfacial adhesion is by adding maleic anhydride polypropylene (MAPP). This coupling agent acts as compatibilizer between the phases by creating bonds. MAPP connects the polar hydroxyl groups from the fiber surface by means of the maleic groups through covalent bonds and hydrogen bonding (Figure 3), with the unmodified PP chains via polymer chain entangling.

**Figure 4:**
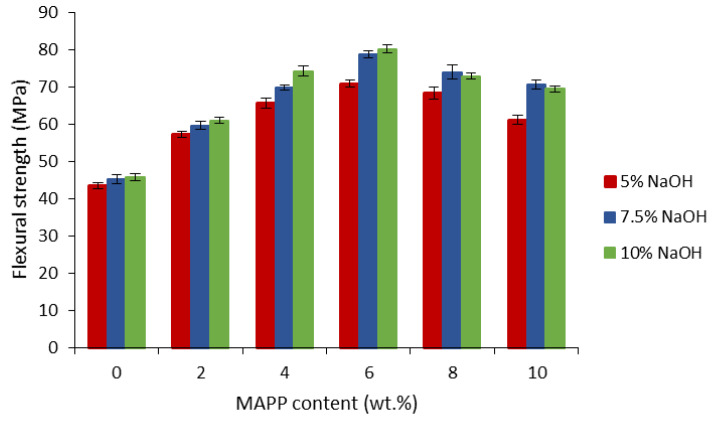
Evolution of the flexural strength of composites with MAPP content.

The effectiveness of the coupling agent depends on the number of bonds and the interaction quality. The literature shows optimal amounts of MAPP needed to enhance the interfacial adhesion in the range of 4–10 wt.% with respect to fiber content [35,36]. In the present work, the amount of MAPP varied from 0 to 10 wt.% with respect to the fiber loading. The influence of MAPP was studied in composites reinforced with a 40 wt.% of hemp core fibers, treated at 5%, 7.5%, and 10% of NaOH. In each case, the flexural strength was measured (Figure 4).

The flexural strength of composites with no coupling agent remained constant and close to the neat matrix values (40.2 MPa), evidencing scarce compatibility between the phases. In fact, this poor compatibility in the lack of the coupling agent is one of the reasons hemp core fibers are commonly used as filling agents rather than reinforcing ones. With the MAPP content, the flexural strength increases until 6 wt.% of MAPP, where the maximum values of the flexural strength are reached. At higher MAPP content, the flexural strength starts to decrease again. This indicates a saturation of fibers’ hydroxyl groups with MAPP molecules, and instead there is self-entanglement of MAPP chains and slippage of the molecules [37,38].

The highest flexural strength is obtained for composites reinforced with hemp core fibers treated with 10% NaOH and containing 6 wt.% of MAPP. By the lignin removal, hydroxyl groups at the fiber surface are more exposed, and thus accessible to the coupling agent. As a result, more bonds are generated between the phases, and thus the strength is increased at the interphase.

### 3.3. Flexural Properties of Hemp Core Fiber Composites

Once the amount of coupling agent was optimized, composite materials at weight ratios of 90/10, 80/20, 70/30, 60/40, and 50/50 (PP/hemp core fibers) were prepared, and the flexural properties measured. In Table 2, the fiber volume fraction (V^f^), the flexural strength of the composites (σ_f_^c^), the deformation at break (ε_f_^c^), and the contribution of the matrix to the flexural strength of the composite (σ_f_^m*^) are presented. The values of σ_f_^m*^ were acquired from the stress–strain curve of polypropylene, by measuring the stress of the matrix at the deformation where the maximum stress of the composite is produced.

**Figure 5:**
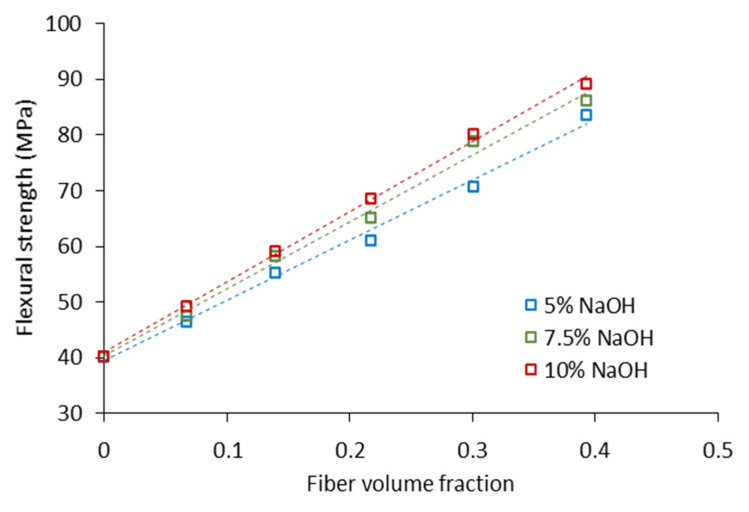
Flexural strength of composites with the fiber volume fraction for each NaOH treatment.

The flexural strength of composites incremented linearly with the fiber content (Figure 5). The highest increments in the flexural strength were perceived in 10% treated fibers. This was expected, owing to the lower lignin content and larger aspect ratios in comparison to the 7.5%- and 5%-treated fibers.

Composites containing 50 wt.% of hemp core fibers showed flexural strength between 2 and 2.3 times higher than the neat matrix, for the hemp core fibers treated at 5–10% of NaOH. These are considered important increments considering that the lignocellulosic material is an industrial residue with low added value. In fact, other lignocellulosic sources from annual plants such as abaca or wood exhibit comparable flexural strengthening [28,34,39]. Briefly, the incorporation of wood fibers to polypropylene returned about 75 and 80 MPa at 40 and 50 wt.% of reinforcement content. Abaca strands exhibited slightly superior reinforcing effect, achieving 87 and 103 MPa at 40 and 50 wt.% of fiber loading. However, this was expected owing to large cellulose contents and high aspect ratios of abaca strands. Overall, the significant potential of hemp core fibers in polymer composites after NaOH treatment for further fiber individualization is suggested.

The linear increase of the flexural strength with the fiber loading suggests a good dispersion of the fibers inside the matrix. In fact, the matrix was charged with up to 50 wt.% of reinforcement. This contrasts with other types of lignocellulosic fibers, particularly highly delignified fibers with abundant hydroxyl groups, where a clear trend of fibers aggregation is observed at elevated fiber loadings. This difficulty is particularly common in composites using polyolefins as polymer matrix, since their high degree of hydrophobicity contrasts very much with the hydrophilic nature of fibers. However, the presence of optimal amounts of lignin, being the most hydrophobic compound in the fiber cell wall, can promote the dispersion of the reinforcement inside the hydrophobic matrix. A recent work highlighted the influence of lignin in enhancing the mechanical properties [11]. In that work, optimal amounts of lignin were proved to secure the stress transferring between the phases promoting the strengthening of the composite. Additionally, other authors suggested the use of lignin as binder between the fibers and hydrophobic matrices [40,41]. In general, there seems to be a balance between the lignin content, which provides a good dispersion of the reinforcement inside the matrix, and the hydroxyl groups in cellulose, ensuring a good interfacial bonding with the coupling agent.

The fact that polymers are able to withstand elevate contents of reinforcements maintaining the linearity of the flexural strength is viewed as an economic and environmental advantage, since it is possible to save part of the matrix maintaining a good performance of the composite [39].

As expected, the increased rigidity of the composites caused by the addition of a stiffer phase also promoted a noticeable reduction in the materials deformation. However, this reduction was not too much in comparison with other natural fiber composites [36].

### 3.4. Modeling the Flexural Strength

One of the most common methods used to compute the contribution of the reinforcement and the matrix to the composite’s strength is by means of the modified Rule of Mixtures (mRoM) [42]. The model establishes a linear relationship which considers the strength supported by the polymer matrix and the stress effectively transferred to the reinforcing fibers. Although the model was originally conceived for modeling tensile properties, the rule may also be applied to flexural properties. Equations (1) and (2) present the mRoM for tensile and flexural strength, respectively.

Tensile mRoM

(1) $$\begin{array}{cc} \mathrm{Tensile}\;\mathrm{mRoM} & \sigma_{t}^{c}=f_{c,t}\cdot \sigma_{t}^{F}\cdot V^{f}+\sigma_{t}^{m^{*}}*\left(1-V^{f}\right) \end{array}$$

(2) $$\begin{array}{cc} \mathrm{Flexural}\;\mathrm{mRoM} & \sigma_{f}^{c}=f_{c,f}\cdot \sigma_{f}^{F}\cdot V^{f}+\sigma_{f}^{m^{*}}*\left(1-V^{f}\right) \end{array}$$

In Equation (1) and (2), $f_{c,t}$ and $f_{c,f}$ are the tensile and flexural coupling factors, respectively, and $\sigma_{t}^{F}$ and $\sigma_{f}^{F}$ are the intrinsic tensile and flexural strength of the fibers, respectively. Generally, in natural fiber composites with strong interfacial bonding, the coupling factors renders values between 0.18 and 0.20 [43].

In both cases, the equations contain two incognita since the determination of the intrinsic strength of the fibers is not feasible by any practical means. The intrinsic tensile strength can be computed by following Kelly and Tyson modified equation [44] and its solution provided by Bowyer and Bader [45], as shown in previous works [46]. Nonetheless, the calculus of the intrinsic flexural strength of the fibers is more confusing. In a recent work, Hashemi [47] assumed the intrinsic flexural and tensile strength to be directly correlated to the flexural and tensile strength of the composite $\left[\sigma_{f}^{F}=\left(\sigma_{f}^{c}/\sigma_{t}^{c}\right)\cdot \sigma_{t}^{F}\right]$. However, as the same author stated, this suggestion might not necessarily suit the reality.

Instead, an alternative methodology allowed the determination of the intrinsic flexural strength. Here, the flexural coupling factor was supposed to be in the same order of magnitude as the tensile coupling factor ($f_{c,f}\equiv f_{c,t})$. This assumption is supported by the fact that the coupling factor, mainly influenced by the efficiency, orientation, and aspect ratio of the fibers [11], should not depend on the type of test conducted. With this in mind, the stress effectively transferred through the reinforcement to the flexural strength ($f_{c,f}\cdot \sigma_{f}^{F}\cdot V^{f})$ and tensile strength ($f_{c,t}\cdot \sigma_{t}^{F}\cdot V^{f})$ of the composite should be directly correlated to the intrinsic flexural ($\sigma_{f}^{F})$ and tensile strength ($\sigma_{t}^{F})$ of the fibers. Thus, the contribution of the fibers to the strength of the composites can be related to its intrinsic strength.

If we represent the difference between the flexural strength of the composite and the contribution of the matrix $\left[\sigma_{f}^{c}-\sigma_{f}^{m^{*}}*\left(1-V^{f}\right)\right]$ with the fiber volume fraction $\left(V^{f}\right)$ of the composites, the slope of the line will determine the contribution of the fibers ($f_{c,f}\cdot \sigma_{f}^{F})$ to the flexural strength of the composites. This factor is defined as the Fiber Flexural Strength Factor (FFSF) in accordance with Thomason methodology [48]. The FFSF factor is described in Equation (3).

(3) $$FFSF=f_{c,\;\;f}\cdot \sigma_{f}^{F}=\left(\frac{\sigma_{f}^{c}-\sigma_{f}^{m^{*}}*\left(1-V^{f}\right)}{V^{f}}\right)$$

By analogy, the mean contribution of the fiber to the tensile strength of the composite can be computed via the Fiber Tensile Strength Factor (FTSF), following Equation (4).

(4) $$FTSF=f_{c,\;\;t}\cdot \sigma_{t}^{F}=\left(\frac{\sigma_{t}^{c}-\sigma_{t}^{m^{*}}*\left(1-V^{f}\right)}{V^{f}}\right)$$

Such factors are considered useful to evaluate the contribution of the fibers to the composite property, since they are independent of the type of matrix used and the fiber loading.

Once the FTSF and FFSF are defined, the intrinsic flexural strength value of hemp core fibers was obtained by using the ratio between the FFSF and FTSF, as shown in Equation (5).

(5) $$\frac{\sigma_{f}^{F}}{\sigma_{t}^{F}}=\frac{FFSF}{FTSF}$$

### 3.5. Hemp Core Fibers Intrinsic Flexural Strength

To determine the mean contribution of the reinforcement to the tensile strength (FTSF), the tensile properties were measured. To this end, standardized specimens were injection molded and tested under tensile forces. From the tensile test, the average and standard deviations values of the composite tensile strength $\left(\sigma_{t}^{c}\right)$, matrix contribution to the flexural strength $\left(\sigma_{t}^{m^{*}}\right)$, and deformation at break $\left(\varepsilon_{t}^{c}\right)$ are collected in Table 3.

**Figure 6:**
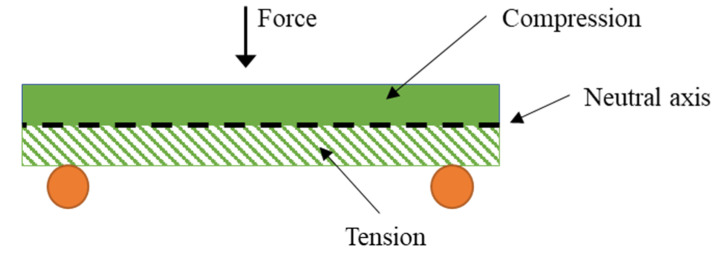
Illustration of the compression and tension loads at the bending test.

Briefly, the tensile strength evolved linearly with the fiber volume fraction. In fact, the tensile strength followed a similar trend to flexural strength. At a 50 wt.%, the tensile strength of the composite was between 1.8 and 2 times higher than the strength of the plain matrix, for NaOH treatments of 5–10%. Nonetheless, the flexural strength values were representatively higher than the tensile ones. This is explained by the fact that composites submitted to bending forces experience a combination of compressive and tensile loads at the cross-sectional area of the specimens (Figure 6).

Most polyolefins, including polypropylene, can support higher loads under compression than under tensile forces. Consequently, materials under flexural loads will be able to withstands higher forces than those submitted to purely tensile forces. Other authors state that the anisotropy of the fibers and their semi-alignment inside the plastic can contribute more extensively to the flexural strength [28].

**Figure 7:**
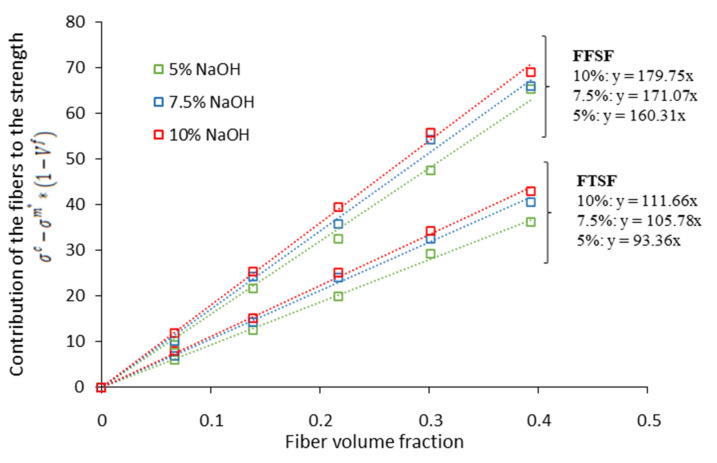
Contribution of the fiber to the composite’s strength at different fiber content.

Overall, one can except higher values of the FFSF than FTSF. The contribution of the fibers computed by means of the FTSF and FFSF are represented in Figure 7.

The values of the FFSF were 179, 171, and 160, whereas the FTSF values were 111, 105, and 93, respectively, for the different NaOH treated fibers.

From the FFSF and FTSF values, the intrinsic tensile strength can be measured. In a previous work [46], the intrinsic tensile strength and tensile coupling factor of hemp core fiber composites was determined (Table 4).

The intrinsic tensile strength of hemp core fibers at different fiber loads can be determined by considering the tensile coupling factor as constant. It is known that the tensile coupling factor results from the product of the length ($X_{1}$) and orientation factor ($X_{2}$) ($f_{c,t}=X_{1}\cdot X_{2})$. The length factor depends on the morphology ($l^{F}/d^{F})$ and the interfacial shear strength (τ), whereas the orientation factor is function on the compounding parameters and the geometry of the mold. In the current study, these factors ($X_{1},\;X_{2}$) were considered constant for every reinforcement amount, as was the tensile coupling factor.

Knowing the tensile coupling factor, the intrinsic tensile strength of the fibers is isolated from the mRoM. Additionally, the ratios between the FFSF and FTSF allow us to compute the intrinsic flexural strength of hemp core fibers (Equation (5)). Subsequently, the flexural coupling factors are computed by means of the flexural mRoM. The results are shown in Table 5.

The mean intrinsic flexural strength of hemp core fibers was 770, 849, and 940 MPa, respectively, for the treatments at 5%, 7.5%, and 10% NaOH concentrations. For the same treatments, the mean intrinsic strength of hemp core fibers was 448, 525, and 586 MPa, respectively.

**Figure 8:**
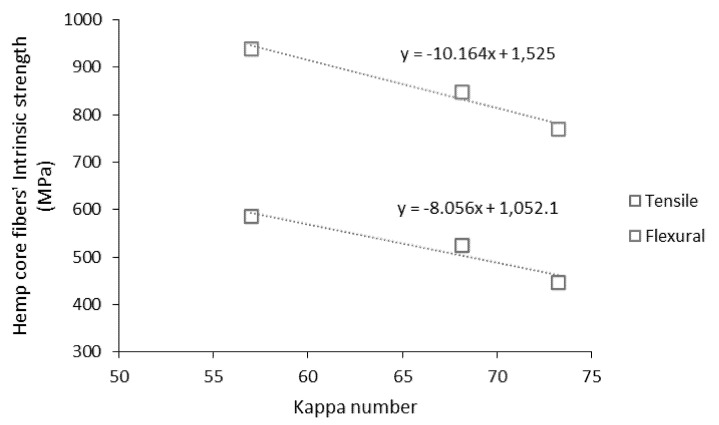
Intrinsic strengths of hemp core fibers with the kappa number.

The intrinsic strength of the fibers under flexural and tensile conditions were dependent on the severity of the NaOH treatment. Therefore, the dependence between the Kappa number, indicative of the lignin content, and the intrinsic strength of the fibers can be stated, as shown in Figure 8.

Both the intrinsic flexural and tensile strengths evolved linearly with the kappa number, with the numerical relation included in Figure 8. In addition, all the mean flexural coupling factors were found between 0.200 and 0.192. These values are comprised in the range of composites with strong interphases. Therefore, the reinforcing ability of the hemp core fibers produced in the present study and the significant influence when tested under flexural conditions can be stated.

## 4. Conclusions

Hemp core residues were chemically treated with sodium hydroxide solutions at different concentrations, and single hemp core fibers were isolated after further mechanical defibering. Composites were produced by loading polypropylene up to 50 wt.% of these hemp core fibers. The effect of the NaOH treatment on the flexural properties of composites and the intrinsic flexural strength of the fibers were investigated. Hemp core fibers behaved as reinforcing elements, increasing by more than two-fold the original flexural strength of the plain matrix. The NaOH treatment promoted the removal of lignin from the cell wall of hemp core biomass. The resulting hemp core fibers showed higher aspect ratio as the NaOH treatment was harsher. Results from the mechanical test reveal increments in the flexural strength similar to those in wood composites. The highest increments were attained in composites with hemp core fibers obtained from 10% NaOH treatment. In all cases, the flexural strength evolved linearly with the fiber content, evidencing good compatibility and dispersion of the two phases.

Besides, a methodology was proposed for the prediction of the intrinsic flexural strength of the fibers. A maximum value was achieved in fibers treated with 10% NaOH (940 MPa), comparable to other sources of natural fibers. Moreover, a clear correlation was observed between the kappa number and intrinsic strength of the fibers. Overall, the investigation opens the possibility and great potential behind the valorization of NaOH treated hemp core residues by its incorporation in polymer composites.

## Figures and Tables

**Table 1:** Manufacturing yield, kappa number and morphology of hemp core fibers.

NaOH	Yield (%)	Kappa Number	l^F^ (µm)	d^F^ (µm)	l^F^/d^F^
5.0%	78.1	73.2 ± 0.3	569 ± 9	23.8 ± 0.4	23.9
7.5%	76.4	68.1 ± 0.3	689 ± 13	24.7 ± 0.3	27.9
10.0%	66.9	57.0 ± 0.4	719 ± 10	24.6 ± 0.3	29.2

l^F^, mean fiber length weighted in length; d^F^, mean fiber diameter.

**Table 2:** Flexural properties of the matrix and composites at different fiber content and NaOH treatment.

6 wt.% MAPP/fiber					
NaOH	Hemp Core	V^f^	σ_f_^c^	σ_f_^m*^	ε_f_^c^
(wt.%)	(wt.%)		(MPa)	(MPa)	(%)
0	0	0	40.20 ± 0.45	40.20 ± 0.45	9.60 ± 0.39
5	10	0.067	46.51 ± 0.59	40.03 ± 0.41	7.89 ± 0.25
	20	0.139	55.21 ± 0.88	38.89 ± 0.49	6.89 ± 0.39
	30	0.217	61.05 ± 0.41	36.39 ± 0.45	5.78 ± 0.33
	40	0.301	70.85 ± 1.03	33.48 ± 0.56	4.90 ± 0.48
	50	0.393	83.59 ± 1.11	30.11 ± 0.50	4.11 ± 0.40
7.5	10	0.067	47.62 ± 0.93	40.15 ± 0.36	8.14 ± 0.26
	20	0.139	58.35 ± 0.82	39.48 ± 0.42	7.26 ± 0.33
	30	0.217	65.12 ± 1.08	37.50 ± 0.50	6.21 ± 0.42
	40	0.301	78.80 ± 0.67	35.27 ± 0.33	5.41 ± 0.25
	50	0.393	86.20 ± 1.14	33.33 ± 0.44	4.86 ± 0.36
10	10	0.067	49.28 ± 1.15	40.06 ± 0.44	7.92 ± 0.56
	20	0.139	49.12 ± 1.03	38.27 ± 0.34	7.12 ± 0.41
	30	0.217	68.70 ± 1.22	37.33 ± 0.31	6.14 ± 0.39
	40	0.301	80.20 ± 0.56	35.08 ± 0.39	5.35 ± 0.44
	50	0.393	89.23 ± 0.79	33.18 ± 0.29	4.82 ± 0.33

**Table 3:** Tensile properties of the matrix and composites at different fiber content and NaOH treatment

6 wt.% MAPP/fiber					
NaOH	Hemp Core	V^F^	σ_t_^c^	σ_t_^m*^	ε_t_^c^
(wt.%)	(wt.%)		(MPa)	(MPa)	(%)
0	0	0	27.60 ± 0.20	27.60 ± 0.20	9.30 ± 0.20
5	10	0.067	31.74 ± 0.45	27.47 ± 0.28	6.84 ± 0.48
	20	0.139	35.92 ± 0.83	27.06 ± 0.33	5.24 ± 0.59
	30	0.217	39.60 ± 0.66	25.19 ± 0.39	3.86 ± 0.61
	40	0.301	45.77 ± 0.89	23.51 ± 0.38	3.12 ± 0.53
	50	0.393	49.30 ± 0.68	21.73 ± 0.45	2.58 ± 0.78
7.5	10	0.067	32.57 ± 1.01	27.48 ± 0.36	6.94 ± 0.49
	20	0.139	37.94 ± 0.55	27.54 ± 0.31	5.74 ± 0.33
	30	0.217	42.90 ± 0.73	26.77 ± 0.49	4.71 ± 0.58
	40	0.301	49.86 ± 0.86	24.74 ± 0.30	3.63 ± 0.26
	50	0.393	54.08 ± 1.11	22.14 ± 0.63	2.69 ± 0.71
10	10	0.067	33.63 ± 0.59	27.42 ± 0.21	6.30 ± 0.19
	20	0.139	38.23 ± 0.47	26.89 ± 0.52	5.10 ± 0.44
	30	0.217	45.27 ± 0.99	25.84 ± 0.61	4.25 ± 0.51
	40	0.301	51.83 ± 0.90	25.15 ± 0.45	3.84 ± 0.35
	50	0.393	57.90 ± 1.02	24.54 ± 0.79	3.58 ± 0.69

**Table 4:** Intrinsic tensile strength and tensile coupling factor of hemp core fibers [46].

6 wt.% MAPP/Fiber			
NaOH	Hemp Core	σ_t_^F^	f_c,t_
(wt.%)	(wt.%)	(MPa)	(MPa)
5	40	472	0.206
7.5	40	548	0.197
10	40	584	0.193

**Table 5:** Intrinsic tensile (σ_t_^F^) and flexural strength (σ_f_^F^) of the hemp core fibers, as well as the tensile (f_c,t_) and flexural (f_c,f_) coupling factors.

NaOH	Hemp Core	FFSF/FTSF	f_c,t_	σ_t_^F^	f_c,f_	σ_f_^F^
(wt.%)	(wt.%)			(MPa)		(MPa)
5	10	1.717	0.206	441	0.180	758
	20			439	0.207	755
	30			443	0.197	761
	40			472	0.194	810
	50			444	0.218	764
7.5	10	1.617	0.197	523	0.179	847
	20			518	0.209	838
	30			512	0.199	829
	40			548	0.203	886
	50			523	0.198	847
10	10	1.610	0.193	622	0.178	993
	20			562	0.202	901
	30			597	0.190	953
	40			584	0.198	936
	50			566	0.193	909
